# Monosaccharide-NAIM Derivatives for D-, L-Configurational Analysis 

**DOI:** 10.3390/molecules16010652

**Published:** 2011-01-17

**Authors:** Chunchi Lin, Chien-Yuan Kuo, Kuo-Shiang Liao, Wen-Bin Yang

**Affiliations:** Genomics Research Center, Academia Sinica, No. 128, Academia Road Section 2, Nankang, Taipei 11529, Taiwan

**Keywords:** enantioseparation, 2,3-naphthalene diamine, D-, L-monosaccharides, ligand-exchange capillary electrophoresis, cyclodextrin

## Abstract

The D-, L-enantiomeric pairs of common monosaccharides (xylose, ribose, rhamnose, arabinose, fucose, glucose, mannose, galactose, *N*-acetylgalactosamine, glucuronic acid and galacturonic acid) were derivatized with 2,3-naphthalenediamine to form the corresponding D-, L-aldo-NAIM derivatives. A simple and facile capillary electrophoretic method was established for sugar composition analysis by simultaneously determining the migration times of these aldo-NAIMs using borate buffer at high pH (100 mM, pH 9.0). The methodology is also applicable to sialic acid (ketose monosaccharides). The quantitation level of the proposed method was in the 10~500 ppm range and the LOD was 1 ppm. The enantioseparation of D, L pairs of aldo-NAIMs were also achieved by using modified sulfated−α−cyclodextrin as the chiral selector in phosphate buffer (300 mM, pH 3.0). In addition, the combination by reductive amination of amino-aldo-NAIM agent and D-, L-enantiomeric pairs of monosaccharides formed a diastereomeric pair for saccharide configuration analysis. Aldo-NAIM derivatives are thus shown to be rapid and efficient agents for analyzing saccharide compositions and configurations with good linearity and short analysis times via capillary electrophoresis.

## 1. Introduction

Capillary electrophoresis has been proven as a powerful separation tool in saccharide analysis [1,2]. Carbohydrates are essential materials in many biological processes [3] and many conjugation methods have been developed by tagging carbohydrates [4,5], but new derivatization methods are required to enable capillary electrophoresis analytical methods. A novel method for the conversion of unprotected and unmodified aldoses into aldo-imidazoles has been developed and applied to the compositional analysis of saccharides by capillary electropheresis (CE) of these derivatives [6]. In continuation of our studies on the rapid transformations of aldoses into their imidazole derivatives using 2,3-naphthalene diamine and catalytic iodine in acetic acid solution [7], we developed a protocol for synthesis of the D-, L-aldo-naphthylimidazoles (aldo-NAIMs) from various D-, L-monosaccharide pairs by direct oxidative condensation of aldoses with aromatic vicinal diamines in the presence of iodine. We also demonstrated that the enantioseparation of D-, L-monosaccharide pairs is facilitated by incorporating the imidazole moiety as a UV and fluorescent dye for CE detection.

**Figure 1:**
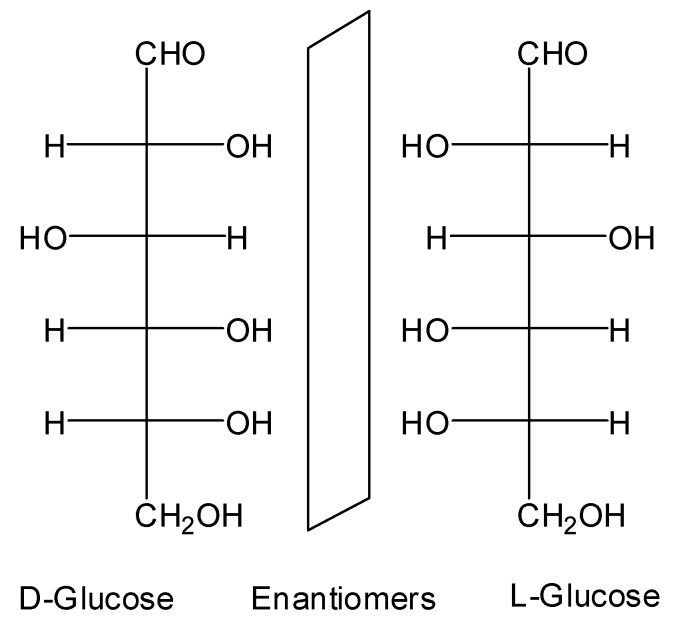
The Fisher projection of D-, L-glucose.

Chiral resolution is an important topic in analytical chemistry [8,9], because chiral enantiomers (Figure 1) have the same physical properties, but may present divergent biological activities. In addition, optical D-,L-monosaccharides presented in Nature lack electric charges and chromophores for chromatographic analysis, making it difficult to separate enantiomeric saccharides by chromatography without some type of chemical derivatization. In recent years CE has proven a good tool to resolve D-, L-monosaccharides [10,11,12,13] and both pre- and in-column introduction of suitable cationic or anionic UV absorbing tags have been used to analyze enantiomeric compounds. 

The use of borate buffers with chiral selectors has been reported for the enantioseparation of saccharides [14] by derivatization with aromatic reagents to label aldoses and these aldoses derivatives were then separated by CE taking advantage of the cavity of cyclodextrins (CDs) to form mixed borate complexes. Stefansson and Novotny first reported the enantioseparation of several monosaccharides by reductive amination with 5-aminonaphthalene-2-sulfonic acid, or 4-amino-5-hydroxynaphthalene-2,7-disulfonic acid, and these derivatized D-, L-polyols were then enantioseparated by CE as complexes with borate using linear or cyclic dextrins as a chiral selector [15]. Kodama *et al.* used the electrophoretic patterns of the condensation of six reducing monosaccharides with PMP. In addition, three monosaccharides, PMP-Man, PMP-Gal, and PMP-Fuc, were enantioseparated by the ligand exchange capillary electrophoresis (LECE) method [16]. In these CE studies, CDs and their derivatives have been widely used as chiral selectors for the enantioseparation of monosaccharides. 

Furthermore, chiral separation by host-guest complexation with CDs in CE analysis can be carried out at different pH values. Phosphate buffer is commonly used at low pH values. For example, heparin, a highly-sulfated glycosaminoglycan, was separated in a chiral mobile-phase for capillary electrophoresis [17]. Kuhn, *et al*. reported enantioseparation of several enantiomers of quinagolides in phosphate buffer (pH 2.5) with CDs as the complexing agent in capillary electrophoresis [18]. The influence of the concentration of the phosphate buffer and pH values on the resolution of enantiomers has been reported. 

Separation and analysis of diastereomers by chromatographic analysis is easier than that of enantiomers. Consequently transformation of enantiomers through a pre-column derivatization to form the corresponding diasteromers is also common practice for the configurational analysis of chiral alcohols [19,20]. Conversion of aldoses by reductive amination at the reducing terminus has been a common practice to tag saccharides for analysis [21]. The *N*-amino-aldo-NAIM moiety appears to be a useful chiral derivatizing agent for the conversion of enantiomeric D-, L-saccharides to diastereomeric products by a common reductive amination. We report herein the conversion of an amino sugar (*N*-Boc-glucosamine) into its aldo-NAIM derivative, and this chiral reagent was then condensed with D-, L-monosaccharides by reductive amination. This represents a new method for saccharide configurational analysis using aldo-NAIM derivitized diastereomers.

## 2. Results and Discussion

**Figure 2:**
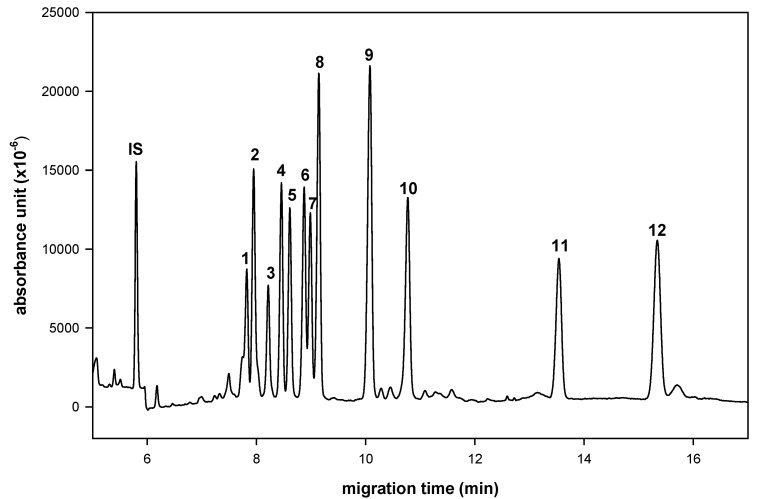
Electrophorogram of 2,3-naphthalene diamine derived monosaccharides. Peaks: 1= Rha-NAIM; 2= GalNAc-NAIM; 3= SA-NAQ (sialic acid-naphthyl benzo[*g*]-quinoxaline); 4= Xyl-NAIM; 5= Rib-NAIM; 6= Glc-NAIM; 7= Man-NAIM; 8= Ara-NAIM; 9= Fuc-NAIM; 10= Gal-NAIM; 11= GlcA-NAIM; 12= GalA-NAIM. Conditions: buffer, 100 mM borate (pH 9.0); applied voltage, 12 kV; uncoated fused-silica capillary, 30 cm × 50 μm I.D.; sample injection, 3s by pressure 0.5 psi; wavelength, 254 nm.

In a preliminary study, we used molecular iodine as catalyst to condense aldoses with aromatic *ortho*-diamines [6,7]. The successful simultaneous separation of 12 aldo-NAIMs occurred when using a Tris buffer in CE for sugar composition analysis. Neutral (rhamnose, xylose, ribose, glucose, mannose, arabinose, fucose, galactose), acidic (*N*-acetylneuraminic acid, glucuronic acid, galacturonic acid) and amino-containing (*N*-acetylgalactosamine) monosaccharides were equally suitable for chemical labeling. Here, we report a simultaneous separation of 12 aldo-NAIMs by using a borate buffer to improve the resolution and eluent running times in CE analysis (Figure 2). The migration times of the aldo-NAIMs are listed in Table 1. Optimization of the separation was achieved by optimizing the CE conditions, including pH value, working temperature, applied voltage and concentration of borate buffer (see Experimental). The migration velocities of derivatives were affected primarily by the extent of aldo-NAIM-borate complexation [16]. The speculative elution order of aldo-NAIM derivatives was assigned by the number of hydroxyl groups and *N*-acetyl groups, e.g., pentoses, *N*-acetylneuraminic acid and *N*-acetylgalactosamine migrated faster than hexoses and glycuronic acids. The elution order of aldohexose-derivatives was also assignable based on the orientation of hydroxyl groups at the C3/C4 position; e.g., Fuc, which possesses *cis*-oriented hydroxyl groups was retarded more than Rha with *trans*-oriented hydroxyl groups. The same behavior could be observed with hexoses and hexuronic acids. Consequently, the compounds listed in their elution order are: Glc (*trans*), Man (*trans*) > Gal (*cis*); Rha (*trans*) > Fuc (*cis*); Xyl (*trans*), Rib (*trans*) > Ara (*cis*) and GlcA (*trans*) > GalA (*cis*). Considering the resolution and speed, the optimized CE conditions were set as 100 mM borate buffer and pH 9.0 at 12 kV and 25 ºC.

**Table 1:** The migration times of 2,3-naphthalene diamine derivated monosaccharides.

Peak	aldo-NAIM	Migration Time (min)
1	Rha-NAIM	7.7
2	GalNAc-NAIM	7.9
3	SA-NAQ	8.2
4	Xyl-NAIM	8.4
5	Rib-NAIM	8.5
6	Glc-NAIM	8.8
7	Man-NAIM	8.9
8	Ara-NAIM	9.1
9	Fuc-NAIM	10.1
10	Gal-NAIM	10.8
11	GlcA-NAIM	13.5
12	GalA-NAIM	15.3

**Scheme 1:**
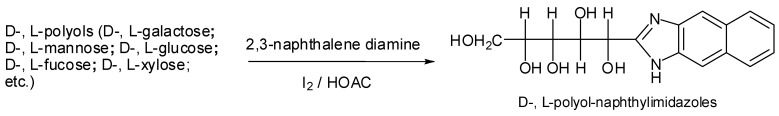
The preparation of D-, L-polyol-NAIMs by a domino reaction of aldose-aromatic *ortho*-diamine (2,3-naphthalene diamine) condensation.

Chiral resolution is a difficult topic in analytical chemistry [8,9], since enantiomers have identical physicochemical properties. The development of suitable methods for enantiomeric separation and quantification was an understandable challenge in the past. In advance, we investigated that if these D-, L-aldo-NAIMs can be used for chiral resolution using the LECE method. According to our previous report [7], we used 2,3-naphthalene diamine to label D-, L-monosaccharides through an iodine catalytic condensation reaction (Scheme 1). 

**Figure 3:**
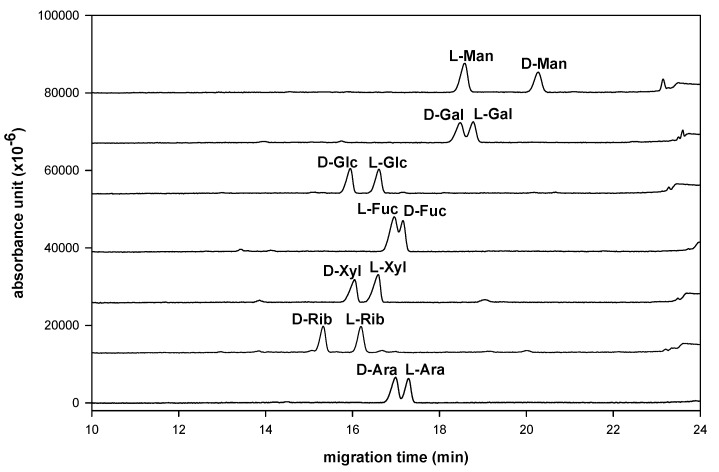
Chiral resolution of seven enantiomeric pairs of naphthalenediamine derivated monosaccharides.

A series of suitable D-, L-polyol-NAIM derivatives were prepared for enantioseparation. This method generated a planar imidazole group through a domino reaction [22]. The synthetic yields are generally excellent, without byproducts, and the reaction times are also short under mild (under 50 °C) and ambient temperatures. Furthermore, we next examined these D-, L-polyol-NAIMs using sulfated−α−CD as the selector ligand in LECE for enantioseparation of monosaccharides. Although we observed that the borate buffers are a good BGE for the compositional analysis of aldo-NAIMs in CE (Figure 2), the enantioseparation ability using the borate buffer system was insufficient to contribute the chiral resolution of D-, L-aldo-NAIMs. Later, we used the phosphate buffer−α−CD system to resolve this question. The enantioseparation of seven kinds of D-, L-monosaccharides is listed in Table 2 and the migration diagrams of D-, L-enantiomer pairs are shown in Figure 3. Each pair of D-, L-monosaccharides was separated, individually. The elution order of D-, L-aldo-NAIM derivatives was also explainable by the orientation of hydroxyl groups at the C3/C4 position that was mentioned in the previous section on compositional analysis. Electrophoretic experiments were set up in 10 mg/mL sulfated−α−CD, 300 mM phosphate buffer at pH 3.0 with an uncoated fused-silica capillary. The capillary temperature was kept at 30 °C and the analytes were detected by their UV absorption at 254 nm. The power supply was operated in the constant-voltage mode, at 15 kV. Each D-, L-monosaccharide pair could be identified even if a short capillary column (30 cm × 50 μm) was used. 

**Table 2:** The migration times of 2,3-naphthalene diamine derivatized D-, L-monosaccharides.

D-enantiomer	Migration time (min)	L-enantiomer	Migration time (min)
D-Manno-NAIM	20.4	L-Manno-NAIM	18.7
D-Galacto-NAIM	18.5	L-Galacto-NAIM	18.9
D-Gluco-NAIM	16.0	L-Gluco-NAIM	16.6
D-Fuco-NAIM	17.2	L-Fuco-NAIM	17.0
D-Xylo-NAIM	16.0	L-Xylo-NAIM	16.6
D-Ribo-NAIM	15.4	L-Ribo-NAIM	16.3
D-Arabino-NAIM	16.9	L-Arabino-NAIM	17.2

In Figure 3, the enantioseparation ability depends strongly on the stability of the ternary complex [9]. It seems that the electrophoretic pattern of aldo–NAIMs depends on ionic strength of the phosphate solution, which affects the running current during the electrophoresis. The longer migration time at the higher ionic concentration may be attributed to an increase in the viscosity, which can increase the chance of interaction between the analyte enantiomers and the sulfated−α−CD complex to give the higher resolution, however, to keep the migration time moderately short, 10 mg/mL sulfated−α−CD with 300 mM phosphate buffer was used and the migration time was shortened down under 20 mins. Consequently, the optimum conditions for enantioseparation as a BGE with both high resolution and moderately short migration time were determined to be 10 mg/mL of sulfated−α−CD with 300 mM phosphate buffer at pH 3.0, 30 °C, 15 kV and 254 nm. To identify the best chiral selector in this experiment several selectors (sulfated−α−CD, sulfated−β−CD, hydroxypropyl–β−CD, 2,6-dimethyl–β−CD and 2,3,6-trimethyl–β−CD) were compared, among which sulfated−α−CD was the best chiral selector based on the resolution ability in this study. Commonly the D-forms migrated faster than the L-forms using the sulfated−α−CD (except for mannose and fucose, Figure 3). The most likely explanation for this migration behavior is that the separation of the enantiomers is due to formation of diastereomeric ternary complexes, however, the detail mechanism of interaction between aldo-NAIM and chiral selector is unclear. 

**Figure 4:**
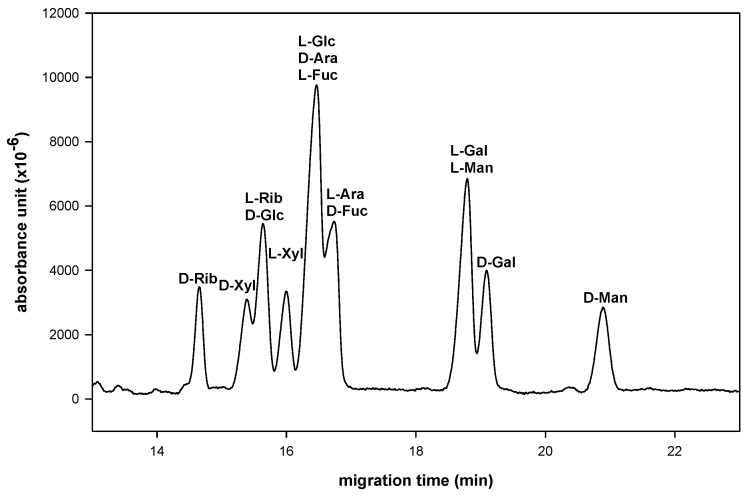
Simultaneously analysis sugar composition and its D-, L-configuration in one inject. Enantioseparation condition was set as phosphate−sulfated−α−CD system with a 30/40.2 cm × 50 µm uncoated fused-silica capillary. (NaH_2_PO_4_, 300 mM, pH 3.0, sulfated−α−CD, 10 mg/mL at 15 kV).

Next simultaneous analysis of several common saccharide pairs of D-, L-aldo-NAIMs was investigated by LECE using phosphate as a central ion with sulfated−α−CD as a chiral selector ligand (Figure 4). Each D-, L-monosaccharide pairs could be clearly identified, even some of peaks were overlapped. This result indicated that LECE was applicable to separate several enantiomeric pairs of monosaccharides in the same time, which is attributed to the formation of a mixture of diastereomeric aldo–NAIM–sulfated−α−CD ternary complexes together with various types of aldo–NAIMs. This method can be applied to the simultaneous sugar composition and D-, L-configuration analysis in the same time for glycosides and saccharides.

**Scheme 2:**
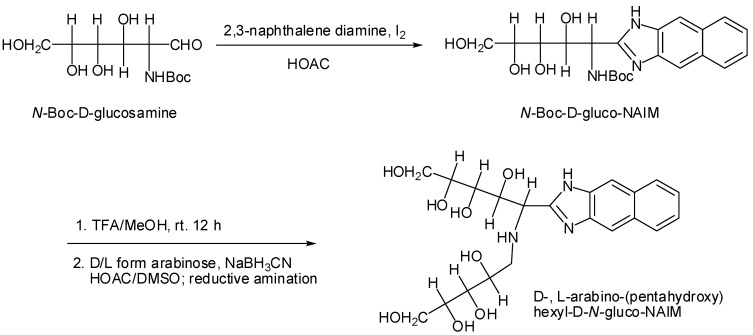
Synthesis of D-, L-arabino-(pentahydroxy)-hexyl-D-*N*-glucosamino-NAIM diastereomers.

Without chiral selector agents it is not possible to separate enantiomeric compounds in an achiral capillary. Therefore, enantiomers were converted to their diastereomers though chemical derivation for chiral resolution. On the other hand, diastereoseparation by chromatographic analysis is easier than enantioseparation. We further prepared aldo-NAIM derivatized diasteromers for enantioseparation of monosaccharides. The strategy and synthetic routes are shown in Scheme 2. First, *N*-Boc-D-glucosamine reacts with 2,3-naphthalenediamine by our previous method to form a *N*-Boc-D-gluco-NAIM, and after deprotection of the *t*-butoxylcarbonyl (Boc) group by TFA for 3 mins [23], the free amino group condensed with D-, L-aldose by reductive amination to generate a new diastereomeric pair of D-aldo-(pentahydroxy)-hexyl-D-*N*-glucosamino-NAIM and L-aldo-(pentahydroxy)-hexyl-D-*N*-glucosamino-NAIM. This method is an alternate way for configurational analysis of aldoses. 

Indeed, the *N*-amino-aldo-NAIM appears to be a useful chiral derivatizing agent for the enantioseparation of enantiomeric D-, L-saccharides in CE analysis. The absolute configuration of the D-, L-aldoses giving rise to chromatographic peaks was unambiguously determined without added chiral selector in the electrophoresis system (e.g., D-arabinose derivative at 6.60 min, L-arabinose derivative at 6.93 min, respectively). We report here a cheap amino-sugar derivative, *N*-Boc-D-gluco-NAIM, is a potent chiral resolution agent for condensing with D-, L-aldoses to form diastereomers by reductive amination for saccharide configurational analysis. The diastereomers of D-, L-aldo-D-*N*-glucosamino-NAIMs can also be monitored by HPLC and TLC methods to facilitate the enantioseparation of aldoses. 

## 3. Experimental

### 3.1. General

A Beckman P/ACE System MDQ (Fullerton, CA, USA) equipped with a filter UV detector and a liquid-cooling device was used for CE analysis. NMR studies, ^1^H/^13^C NMR experiments were performed on a Bruker Fourier transform spectrometer (AV-600) equipped with a 5 mm DCI dual cryoprobe. Spectra were obtained at 298 K in DMSO-*d_6_*. The molecular weights of the proton adducts of ions [M + H]^+^were determined using a ESI-TOF MS (Bruker Bio-TOF III).

### 3.2. Chemicals and reagents

D, L-Glucose (Glc), D, L-galactose (Gal), D, L-mannose (Man), D, L-arabinose (Ara), D, L-ribose (Rib), D, L-xylose (Xyl), D, L-fucose (Fuc), rhamnose (Rha), *N*-acetyl galactosamine (GalNAc), *N*-Boc glucosamine (*N*-Boc-Glc), glucuronic acid (GlcA), sialic acid (SA), sulfated α-cyclodextrin (sulfated α-CD) and 2-naphthol (used as the internal standard, IS) were purchased from Sigma-Aldrich (St. Louis, MO, USA). Galacturonic acid (GalA) was obtained from Fluka (Buchs, Switzerland). 2,3-Naphthalene diamine, methyl sulfoxide (DMSO), sodium borohydride (NaBH_4_), acetic acid and iodine were purchased from Acros (New Jersey, USA). Disodium tetrabrate, sodium dihydrogen phosphate, hydrochloric acid (HCl) and sodium hydroxide (NaOH) were obtained from Merck (Darmstadt, Germany) and all materials are analytical grade. Milli-Q water (Millipore, Bedford, MA, USA) was used for the preparation of buffer and related aqueous solution.

### 3.3. Preparation of aldo-NAIMs

A solution of aldose (1.8 mg, 1.0 μmol) in AcOH/H_2_O (v/v = 10:1, 1.5 mL) was treated with an 2,3-naphthalenediamine tag (1.6 mg, 1.0 μmol) and iodine (2.0 mg, 0.8 μmol). The reaction mixture was stirred at room temperature for 6~18 h to completion, as indicated by the TLC analysis. The mixture was concentrated under reduced pressure, and then diluted with water and triturated with EtOAc. The organic layer was removed, and the residue was concentrated in vacuo to give the crude product of aldo-NAIM (2.5 mg, 76% yield). The aldo–NAIM products were directly determined by CE analysis without further purification. For preparation of *N*-amino-aldo–NAIM derivatived diastereomers, the *N*-Boc-D-glucosamine (278.1 mg, 1.0 mmol) was reacted with 2,3-naphthalenediamine (174 mg, 1.0 mmol) with our previous method to form a *N*-Boc-D-gluco-NAIM (292.2 mg, 70%), and the *t*-butoxylcarbonyl (Boc) group was removed by TFA for 3 mins to give *N*-amino-gluco–NAIM (52%). This chiral amino agent (3.2 mg, 1.0 μmol) was condensed with D-/L-arabinose (1.6 mg, 1.0 μmol) by reductive amination to generate a new diastereomeric pair of D-/L-arabino-D-*N*-glucosamino-NAIM for chromatographic analysis.

### 3.4. CE system

A Beckman P/ACE System MDQ (Fullerton, CA, USA) equipped with a filter UV detector and a liquid-cooling device was used. The separation of twelve aldo-NAIMs was carried out in an uncoated-silica capillary (Polymicro Technologies, AZ, USA), 40.2 cm (30 cm effective length) × 50 μm I.D. The background electrolyte (BGE) was borate buffer (100 mM, pH 9.0) and samples were injected by pressure (0.5 psi for 3s) at the anodic end of the capillary with a constant voltage of +12 kV. For enantioseparation of seven pairs of enantiomeric aldo-NAIMs, the analyses were achieved by using BGE consisted of phosphate buffer (300 mM, pH 3.0) and sulfated-α-CD (10 mg/mL) as the chiral selector in a 30 cm capillary (effective length) at +15 kV. After CE analysis the each new sample running was conditioned with water for 5 mins and BGE for another 5 mins.

### 3.5. Optimization of the separation of 12 aldo-NAIM derivatives

**Figure 5:**
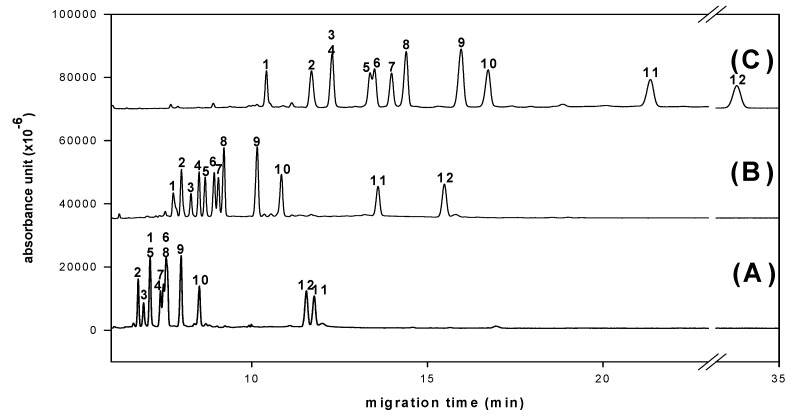
Effects of pH value of borate buffer on derivated monosaccharides separation. (A) pH 8.0 (B) pH 9.0 (C) pH 10.0. Peaks: 1= Rha-NAIM; 2= GalNAc-NAIM; 3= SA-NAQ; 4= Xyl-NAIM; 5= Rib-NAIM; 6= Glc-NAIM; 7= Man-NAIM; 8= Ara-NAIM; 9= Fuc-NAIM; 10= Gal-NAIM; 11= GlcA-NAIM; 12= GalA-NAIM. An uncoated fused-silica capillary (30/40.2 cm × 50 μm) with borate buffer (100 mM), applied voltage (12 kV), separation temperature (25 °C) and 40 ppm samples were used.

Simultaneous determination of twelve aldo-NAIMs was achieved with optimized CE conditions (Figure 2). Effects of pH value and concentration of borate buffer were studied to decide the optimal separation conditions. The resolution and migration time of all derivatives increased in expectation with increasing of pH value (Figure 5, pH 10.0 has good resolution but the migration time is long). 

**Figure 6:**
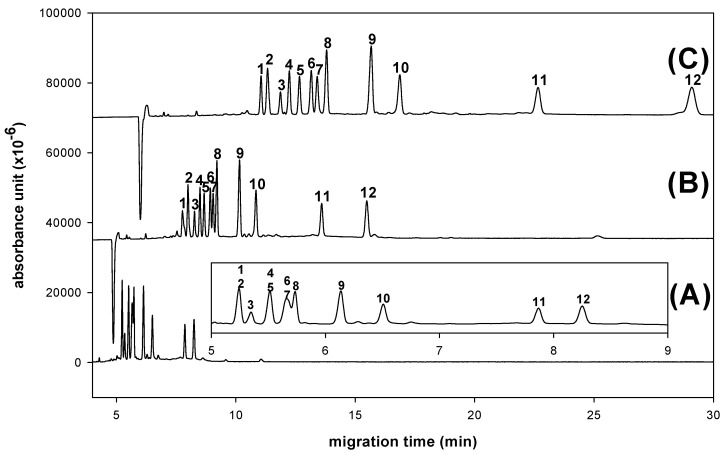
Effects of concentration of borate buffer on derivated monosaccharides separation. (A) 50 mM (B) 100 mM (C) 200 mM. Peaks: 1= Rha-NAIM; 2= GalNAc-NAIM; 3= SA-NAQ; 4= Xyl-NAIM; 5= Rib-NAIM; 6= Glc-NAIM; 7= Man-NAIM; 8= Ara-NAIM; 9= Fuc-NAIM; 10= Gal-NAIM; 11= GlcA-NAIM; 12= GalA-NAIM. An uncoated fused-silica capillary (30/40.2 cm × 50 μm) with borate buffer (pH 9.0), applied voltage (12 kV), separation temperature (25 °C) and 40 ppm samples were used.

The resolution and migration time of all derivatives increased as expected with increasing borate concentration, as shown in Figure 6. The reason for this depends on the destabilization of aldo-NAIM-borate complex. With the high-pH BGE system, the extents of proton ionization of aldo-NAIMs and charge/mass ratio would result in the difference of migration velocities. Considering the resolution and speed, the optimizing CE condition was set as 100 mM borate buffer and its pH was 9.0.

### 3.6. Validation of experimental data

The quantitative determination of aldo-NAIMs was validated. Calibration curves were obtained by the corrected peak area ratio of each aldo-NAIM derivative to internal standard (IS) as ordinate (*y*) versus the concentration of each aldo-NAIM derivative as abscissa (*x*). The precision and accuracy of the intra-day (n = 3) and inter-day (n = 5) analysis were evaluated by analyzing three different concentrations of each aldo-NAIM. Five different concentrations of aldo-NAIMs were analyzed using 2-naphthol as internal standard (IS). The *r^2^* values (>0.9969) of the regression curves indicated high linearity in intra- and inter-day analysis. The relative standard derivation (RSD) and relative error (RE) were calculated to estimate the precision and accuracy of the proposed method by analyzing three different concentrations. The RSD and RE values in intra- and inter-day analysis were less than 8.2% and 9.1%, respectively. The results represented good reproducibility and reliability. The limitations of determination (LOD) were assessed precisely; likewise 1 ppm for Ara and Fuc; 2.5 ppm for GlcA and GalA; 5 ppm for sialic acid and 2 ppm for the others.

*tert-Butyl-(2S,3R,4R,5R)-2,3,4,5-tetrahydroxy-1-(1H-naphtho[2,3-d]imidazol-2-yl)pentylcarbamate* (***N*-Boc-D-gluco-NAIM**). ^1^H-NMR (DMSO-*d_6_*, 600 MHz) *δ* 8.29 (2 H, s), 8.17 (2 H, s), 7.56 (2 H, s), 5.14 (1 H, s), 4.28 (1 H, s), 3.56 (1 H, d, *J* = 10.1 Hz), 3.49 (1 H, d, *J* = 6.1 Hz), 3.40 (1 H, dd, *J* = 10.9, 5.1 Hz), 3.30 (1 H, d, *J* = 7.9 Hz), 1.39 (9 H, s). ^13^C-NMR (DMSO-*d_6_*, 150 MHz) *δ* 170.8, 157.6, 156.9, 133.5, 131.0, 130.5, 128.5, 128.3, 128.2, 126.1, 124.5, 124.2, 111.9, 71.2, 70.4, 70.3, 63.7, 53.0, 52.0, 28.5. HRMS (ESI) calcd for C_21_H_27_N_3_O_6_: 418.1974; found: *m/z* 418.1973 [M + H]^+^.

*(2S,3R,4R,5R)-2,3,4,5-tetrahydroxy-1-(1H-naphtho[2,3-d]imidazol-2-yl)pentylamine* (**D-glucosamino-NAIM**). ^1^H-NMR (DMSO-*d_6_*, 600 MHz) *δ* 8.13 (2 H, s), 8.03 (2 H, dd, *J* = 6.2, 3.4 Hz), 7.40 (2 H, dd, *J* = 6.2, 3.4 Hz), 4.65 (1 H, d, *J* = 7.9 Hz), 4.40 (1 H, d, *J* = 8.3 Hz), 3.56 (1 H, dd, *J* = 11.5, 2.9 Hz), 3.37 (1 H, dd, *J* = 11.5, 2.9 Hz), 3.09 (1 H, d, *J* = 8.4 Hz). ^13^C-NMR (DMSO-*d_6_*, 150 MHz) δ 159.8, 154.2 (2 ×), 130.4 (2 ×), 128.3 (2 ×), 124.1 (2 ×), 118.6, 116.7, 71.0, 70.8, 63.6, 63.5, 52.4. HRMS (ESI) calcd for C_16_H_19_N_3_O_4_: 318.1448; found: *m/z* 318.1459 [M + H]^+^.

*(2’S,3’R,4’R,5’R)-5-(1H-naphtho[2,3-d]imidazol-2-yl)-5-(2R,3R,4R)-(2,3,4,5-tetrahydroxypentyl-amino)pentane-1,2,3,4-tetraol* (**D-arabino-N-D-glucosamino-NAIM**).^ 1^H-NMR (DMSO-*d_6_*, 600 MHz) *δ* 7.98 (2 H, s), 7.95 (2 H, dd, *J* = 6.1, 3.4 Hz), 7.35 (2 H, dd, *J* = 6.1, 3.4 Hz), 4.17 (1 H, d, *J* = 8.1 Hz), 3.67 (2 H, d, *J* = 8.5 Hz), 3.63-3.60 (6 H, m), 3.47-3.35 (4 H, m). ^13^C-NMR (DMSO-*d_6_*, 150 MHz) *δ* 157.9, 130.1 (2 ×), 128.2 (2 ×), 123.8 (2 ×), 120.5, 118.5, 116.5, 114.5. 71.5, 71.4, 71.1, 70.7, 69.5, 63.8, 63.7, 53.1, 52.7. HRMS (ESI) calcd for C_21_H_30_N_3_O_8_: 452.2027; found: *m/z* 452.2012 [M + H]^+^. 

*(2’S,3’R,4’R,5’R)-5-(1H-naphtho[2,3-d]imidazol-2-yl)-5-(2S,3S,4S)-(2,3,4,5-tetrahydroxypentyl-amino)pentane-1,2,3,4-tetraol* (**L-arabino-*N*-D-glucosamino-NAIM**).^ 1^H-NMR (DMSO-*d_6_*, 600 MHz) *δ* 7.97 (4 H, s), 7.35 (2 H, s), 4.44-4.24 (2 H, m), 3.82-3.39 (9 H, m), 3.27 (2 H, br). ^13^C-NMR (DMSO-*d_6_*, 150 MHz) *δ* 159.1 (2 ×), 130.2 (2 ×), 128.3 (2 ×), 124.4 (2 ×), 123.8, 123.3, 116.7, 72.0, 71.3, 71.2, 70.1, 69.0, 68.2, 64.1, 63.5, 49.3, 46.1. HRMS (ESI) calcd for C_21_H_30_N_3_O_8_: 452.2027; found: *m/z* 452.2055 [M + H]^+^.

## 4. Conclusions

We have reported a simple, efficient and environmentally friendly process for labeling saccharides using iodine as a catalyst. Various aldoses react readily with aromatic diamines in acetic acid solution to form the corresponding aldo-imidazoles for compositional analysis. In contrast to the parent saccharides, the aldo-imidazoles have a chromophore and stable molecular structure in CE analysis. In comparison with reductive amination of saccharides, this reaction is easier to operate and environmentally friendly. We also demonstrated that the enantioseparation of D-, L-monosaccharides is facilitated by incorporating sulfated−α−CD as a chiral selector in phosphate buffer (300 mM, pH 3.0). Seven kinds of D-, L-pairs of aldo-NAIMs can be enantioseparated individually in this LECE system (Figure 3). These aldo-NAIMs have a planar imidazole-naphthalene moiety and are also stable at both high and low pH values. The rigid NAIM moiety might help the separation ability of aldo-NAIMs. The present CE method also allows simultaneous chiral resolution of several enantiomeric pairs of monosaccharides (Figure 4). CE provides a rapid method for identification of saccharides’ D-, L-configuration, even when sample is less than 0.01 μmol (2 ppm). We also reported a combination of amino-D-aldo-NAIM and D-, L-enantiomeric monosaccharide by reductive amination to form a diastereomeric pair of D-, L-*N*-arabino-D-gluco-NAIM for saccharide configurational analysis. These methods are promising in further application to investigate the composition and stereo-configuration of saccharides in medicinal herbs. 
